# RETRACTED: Li et al. FBXW7 Acts as an Independent Prognostic Marker and Inhibits Tumor Growth in Human Osteosarcoma. *Int. J. Mol. Sci.* 2015, *16*, 2294–2306

**DOI:** 10.3390/ijms27093781

**Published:** 2026-04-24

**Authors:** Zhanchun Li, Jie Xiao, Kongzu Hu, Gang Wang, Maoqiang Li, Jidong Zhang, Guangqi Cheng

**Affiliations:** 1 Department of Orthopaedic Surgery, Renji Hospital, School of Medicine, Shanghai Jiaotong University, Shanghai 200127, China; shrenji@gmail.com (Z.L.); wly1618@gmail.com (G.C.); 2 Department of Anesthesiology, Renji Hospital, School of Medicine, Shanghai Jiaotong University, Shanghai 200127, China; applexiaomz@gmail.com; 3 Department of Orthopaedic Surgery, The First Affiliated Hospital of Anhui Medical University, Hefei 230022, China; kongzhuhu@gmail.com; 4 Department of Orthopaedic Surgery, The Second Affiliated Hospital of Nanjing Medical University, Nanjing 210029, China; kcb069js@gmail.com; 5 Department of Orthopaedic Surgery, Hangzhou First People’s Hospital, Hangzhou 310006, China; maotai5488@gmail.com

The journal retracts the article “FBXW7 Acts as an Independent Prognostic Marker and Inhibits Tumor Growth in Human Osteosarcoma” [1], cited above.

Following publication, concerns were brought to the attention of the Editorial Office regarding a number of irregularities found in images presented in this article [1].

Adhering to our standard procedure, an investigation was conducted by the Editorial Office and Editorial Board, which confirmed indications of inappropriate image editing regarding an overlap between Figures 3D and 5 published in [1] and Figures 1A and 6B published in [2]; between Figure 3D in [1] and Figure 2D presented in [3] and Figure 4C [4]; between Figures 4B,C and 5 published in [1] and Figures 4B and 5B published in [5]; and between Figure 4A in [1] and Figure 4A published in [6], all showing different experimental conditions. As a result, the Editorial Board has lost confidence in the reliability of the findings and decided to retract this publication [1], as per MDPI’s retraction policy (https://www.mdpi.com/ethics#_bookmark30, accessed on 29 March 2026).

This retraction was approved by the Editor-in-Chief of *IJMS*.

The authors did not provide a comment on this decision.
